# Ancient Sturgeons Possess Effective DNA Repair Mechanisms: Influence of Model Genotoxicants on Embryo Development of Sterlet, *Acipenser ruthenus*

**DOI:** 10.3390/ijms22010006

**Published:** 2020-12-22

**Authors:** Ievgeniia Gazo, Roman Franěk, Radek Šindelka, Ievgen Lebeda, Sahana Shivaramu, Martin Pšenička, Christoph Steinbach

**Affiliations:** 1 South Bohemian Research Center of Aquaculture and Biodiversity of Hydrocenoses, Faculty of Fisheries and Protection of Waters, University of South Bohemia in Ceske Budejovice, Zátiší 728/II, 389 25 Vodňany, Czech Republic; franek@frov.jcu.cz (R.F.); ilebeda@frov.jcu.cz (I.L.); sahana.s92@gmail.com (S.S.); psenicka@frov.jcu.cz (M.P.); steinbach@frov.jcu.cz (C.S.); 2 Laboratory of Gene Expression, Institute of Biotechnology—Biocev, Academy of Science of the Czech Republic, 252 50 Vestec, Czech Republic; radek.sindelka@ibt.cas.cz

**Keywords:** sturgeon, embryo, genotoxicity, DNA damage repair, H2AX, ATM

## Abstract

DNA damage caused by exogenous or endogenous factors is a common challenge for developing fish embryos. DNA damage repair (DDR) pathways help organisms minimize adverse effects of DNA alterations. In terms of DNA repair mechanisms, sturgeons represent a particularly interesting model due to their exceptional genome plasticity. Sterlet (*Acipenser ruthenus*) is a relatively small species of sturgeon. The goal of this study was to assess the sensitivity of sterlet embryos to model genotoxicants (camptothecin, etoposide, and benzo[a]pyrene), and to assess DDR responses. We assessed the effects of genotoxicants on embryo survival, hatching rate, DNA fragmentation, gene expression, and phosphorylation of H2AX and ATM kinase. Exposure of sterlet embryos to 1 µM benzo[a]pyrene induced low levels of DNA damage accompanied by ATM phosphorylation and *xpc* gene expression. Conversely, 20 µM etoposide exposure induced DNA damage without activation of known DDR pathways. Effects of 10 nM camptothecin on embryo development were stage-specific, with early stages, before gastrulation, being most sensitive. Overall, this study provides foundational information for future investigation of sterlet DDR pathways.

## 1. Introduction

DNA is subject to constant damage from endogenously produced reactive oxygen species that affect replication and transcription and can alter its structure [1]. Further, exogenous factors, such as radiation or pollutants, induce DNA damage via oxidation, DNA adduct formation, and single- or double-strand breaks (SSB or DSB, respectively). Cellular mechanisms for repair of DNA damage are highly conserved among vertebrates; however, species-specificity in processes and targets of DNA damage repair (DDR) are noted [2]. Previous studies of fish describe primary DDR pathways: base and nucleotide excision repair (BER and NER), homologous recombination (HR), and non-homologous end-joining (NHEJ) [3,4].

Cells initiate damage recognition and assembly of response complexes in response to DNA damage. An initial signal for DDR response is the phosphorylation of the histone H2A subtype, termed H2AX, at Ser139 [5]. Phosphorylated H2AX is termed γ-H2AX. Several phosphoinositide 3-kinase-related protein kinases are essential for activating cell cycle checkpoints following DNA damage, such as ATM (ataxia telangiectasia mutated) kinase [6,7]. Briefly, ATM is activated by autophosphorylation at Ser1981 which leads to dissociation of inactive ATM dimers into single protein molecules with increased kinase activity [5,8]. ATM initiates phosphorylation of numerous substrates, including H2AX, and 53BP1, as well as checkpoint proteins. These processes stop progression through the cell cycle and activate proteins responsible for DDR [5]. The 53BP1 protein is used as a marker of DNA damage in fish [9]. This protein is a key component of DDR and mediates activation of DSB repair. The protein also exhibits interaction surfaces for numerous DSB-responsive proteins [10].

Genotoxicants are compounds that alter the chemical structure of DNA and induce different types of DNA damage. Benzo[a]pyrene (BaP) is a model polycyclic aromatic hydrocarbon and a ubiquitous contaminant generated from incomplete combustion of organic material. The chemical is also among the most studied contaminants in ecotoxicology [11]. BaP can alter DNA methylation state and displays teratogenic effects on zebrafish embryos with and EC_50_ 0.52 µM at three days post-fertilization [12,13]. BaP metabolites form DNA adducts that promote carcinogenesis [14]. Another well-studied group of model genotoxicants are inhibitors of topoisomerases that are often used in cancer therapy. DNA topoisomerases regulate the topological state of DNA by creating short breaks in the DNA double-helix [15]. Inhibitors of DNA topoisomerase I (e.g., camptothecin, CPT) and II (etoposide) are effective antitumor drugs. Effect of these inhibitors on fish embryo development was previously studied in zebrafish [16]. One study in sterlet evaluated effect of genotoxic PCB on embryo survival and morphometric alterations [17]. However, little information is currently available about genotoxicant impacts on sterlet embryo development.

Sturgeons (belonging to the family Acipenserids) are an attractive model for studying DDR. They display one of the highest numbers of chromosomes, ~120 to ~360, among vertebrates as a result of multiple rounds of lineage-specific whole-genome duplication [18]. Sturgeons are often referred to as “living fossils.” The group likely evolved approximately 200 million years ago [19] making them evolutionarily highly successful. Further, sturgeons have extraordinary genomic plasticity demonstrated through intraspecific hybridization of individuals with different chromosome numbers [20]. Intriguingly, hybridization between intermediate hybrids and pure species gives not only viable progeny but even fertile fish [20]. Sturgeons are also prone to spontaneous polyploidization [21,22]. The basis of this evolutionary success and mechanisms of genome plasticity are still poorly understood; however, such plasticity would require highly effective DNA repair.

Embryonic stages are the most sensitive to DNA damage since DNA replication rate is high, cell proliferation is rapid, and the cell cycle is much shorter compared to adult cells [23]. The integrity of the genome is thus at greater risk, and efficient DNA repair is of significant importance. Sturgeon embryogenesis is described by several authors [24,25]; however, nothing is known about DDR capacity in developing sturgeon embryos. Therefore, we analyzed the effect of genotoxicants, (BaP, camptothecin (CPT), and etoposide) on developing embryos of sterlet (*Acipenser ruthenus*) at different embryonic stages. We evaluated DNA damage caused by these compounds and their adverse effects on sturgeon hatching success and embryonic development. Further, we assessed responses to DNA damage induced by genotoxicants in embryos.

## 2. Results

### 2.1. The Effect of Genotoxicants on Sterlet Embryo Development

**Figure 1:**
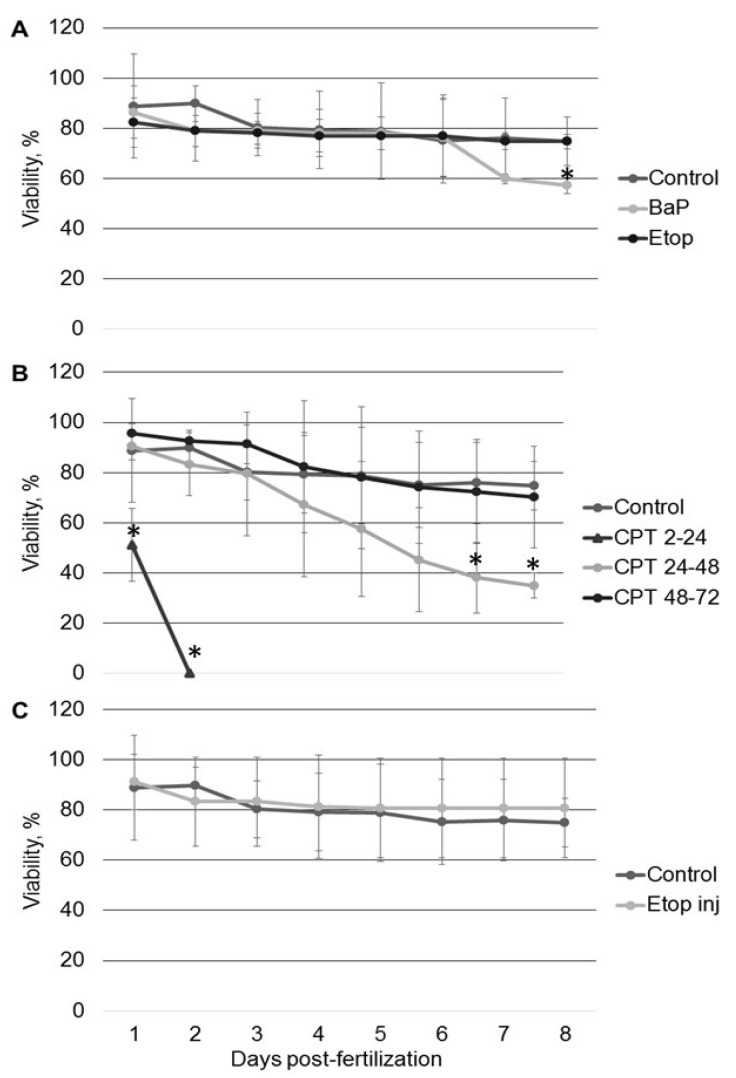
Sterlet embryo viability following exposure to model genotoxicants: (**A**) embryos were exposed to BaP and etoposide from 2 hpf until 8 dpf; (**B**) embryos were exposed to camptothecin (CPT) from 2–24, 24–48, and 48–72 hpf; (**C**) embryos were injected with etoposide at the 1-cell stage. Embryo mortality was monitored daily for 8 days. Results represent the mean of six independent experiments. Error bars represent standard error of the mean. Asterisks indicate significant differences compared to control at the same time point (* *p* < 0.05, Kruskal–Wallis test).

We exposed sturgeon (*Acipenser ruthenus*) embryos to 1 µM BaP, 20 µM etoposide, and 10 nM CPT to assess impacts on viability and hatching rate. Sterlet embryos were exposed to BaP and etoposide continuously from the 2-cell stage until 8 days post-fertilization (dpf), and to CPT for 24 h at different stages of development. Preliminary results showed that short-term exposure to BaP and etoposide at different stages had no effect on embryo viability or hatching rate. Long-term exposure to BaP, but not etoposide, significantly increased embryo mortality. Embryo viability was 57 ± 3%, compared to etoposide-exposed (74 ± 2.8%) and control (75 ± 8%) embryos at 8 dpf (Figure 1A). Conversely, exposure to CPT for 24 h during early developmental stages induced 100% mortality (Figure 1B). Exposure at 24–48 h post-fertilization (hpf) (gastrulation) significantly decreased embryo viability to 34 ± 5% at 8 dpf. In contrast, exposure to CPT at neurulation stage (48–72 hpf) did not significantly affect viability.

Differences in mortality between topoisomerase I and II inhibitors could be attributed to different penetration through the embryo chorion. Therefore, to ensure the proper delivery of etoposide, we microinjected it directly into embryo animal poles. However, no changes were observed in mortality compared to controls (Figure 1C).

**Figure 2:**
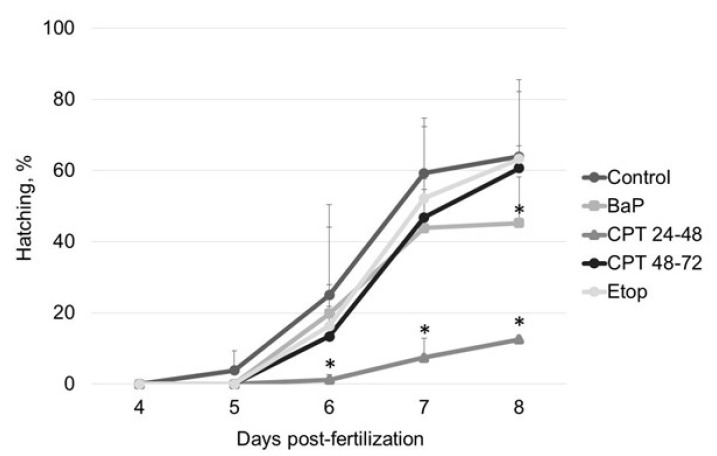
Sterlet embryo hatching rate following exposure to BaP, etoposide, and CPT. Embryos were exposed to BaP and etoposide from 2 hpf until 8 dpf, and to CPT from 2–24, 24–48, or 48–72 hpf. Results represent the mean of six independent experiments. Error bars represent standard error of the mean. Asterisks indicate significant differences compared to the control value at the same time point (* *p* < 0.05, Kruskal–Wallis test).

Exposure to etoposide and CPT at neurulation (48–72 hpf) did not affect the hatching rate at 8 dpf (Figure 2). Initial hatching occurred at 5 dpf in control embryos but was delayed to 6 dpf in treated embryos. Exposure to BaP and CPT at 24–48 hpf significantly decreased hatching success to 45 ± 5% and 13 ± 1.5%, respectively, at 8 dpf.

**Figure 3:**
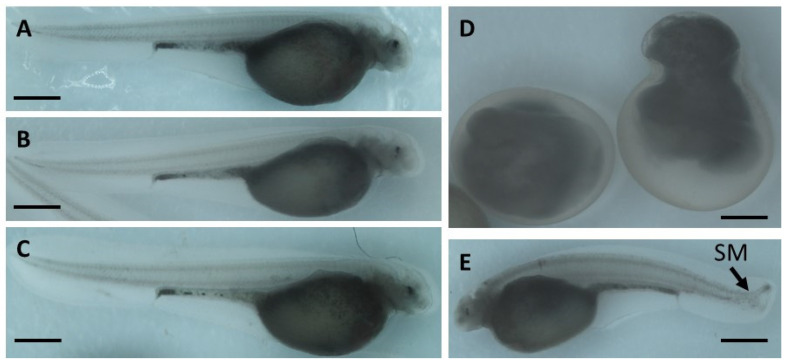
Sterlet embryo phenotypes observed at 8 dpf: (**A**) control; (**B**) etoposide 20 µM; (**C**) BaP 1 µM; (**D**) CPT 10 nM at 24–48 hpf; (**E**) CPT 10 nM at 48–72 hpf. Images are representative of most embryos. Arrow indicates spinal malformation (SM). Scale bar is 1 mm.

Hatched embryos did not show malformations following exposure to BaP and etoposide (Figure 3A–C). However, embryos exposed to CPT at 24–48 or 48–72 hpf exhibited skeletal malformations and a phenotype similar to developmental delay (Figure 3D,E).

### 2.2. DNA Damage Induced by Genotoxicants in Sterlet Embryo

**Figure 4:**
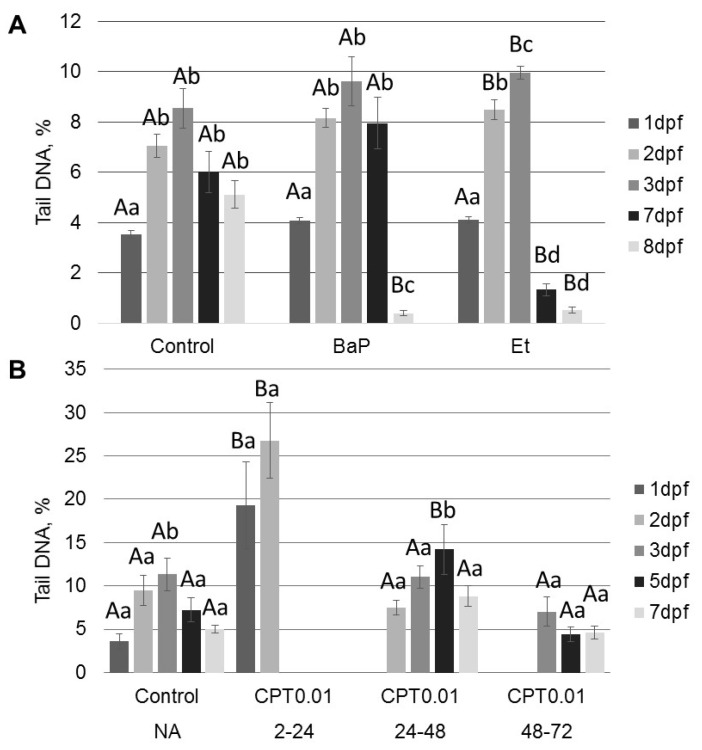
Percent DNA in comet tails was analyzed following exposure to 1µM BaP, 20 µM etoposide (Et), or 10 nM CPT. Embryos were exposed to: (**A**) BaP and etoposide continuously from fertilization to 8 dpf; (**B**) CPT for a short term at 2–24, 24–48, or 48–72 hpf. Percent DNA in comet tails was analyzed in embryos from all groups at 1–8 dpf. Results represent the mean of six independent experiments. Error bars represent standard error of the mean. Capital letters indicate significant difference from the control on the same dpf (analysis of variance (ANOVA), *p* < 0.05). Lowercase letters indicate significant differences among time points (dpf) for each treatment (ANOVA, *p* < 0.05).

We used comet assays to analyze the effects of BaP and topoisomerase inhibitors on DNA integrity in sterlet embryos at different stages. DNA damage increased even in control embryos during development from 3.5 ± 0.1% Tail DNA at 1 dpf to 11 ± 1.9% Tail DNA at 3 dpf (Figure 4). However, DNA integrity was partially recovered to 5 ± 0.6% Tail DNA at 8 dpf. Similarly, an increase in DNA fragmentation was observed at 2–3 dpf in BaP and etoposide treated embryos, followed by recovery at 7 and 8 dpf (Figure 4A). Exposure to 20 µM etoposide induced significantly more DNA damage at 2 and 3 dpf compared to the controls (*p* < 0.05, analysis of variance (ANOVA)). Interestingly, DNA fragmentation level at 8 dpf was lower in embryos exposed to BaP (0.4% ± 0.1%) and etoposide (0.5% ± 0.1%) than in control embryos.

Exposure of sterlet embryos to 10 nM CPT from 2–24 hpf induced a significant increase in DNA damage (19 ± 5% Tail DNA) at 24 hpf (Figure 4B). The level of DNA fragmentation further increased at 2 dpf, reaching 27 ± 4%. Embryos did not survive this level of damage (Figure 1B). Conversely, exposure to the same concentration of CPT at later stages (24–48 and 48–72 hpf) did not induce similar levels of DNA fragmentation (Figure 4B). Embryos exposed to CPT during gastrulation (24–48 hpf) showed significantly higher DNA damage only after 5 dpf (14 ± 2.9%) compared to the control (7 ± 1.3%) and this DNA fragmentation was partially repaired by 7 dpf (9 ± 1.2%). Comet assays did not detect significant differences in DNA integrity between control embryos and embryos treated with CPT at neurulation (48–72 hpf). This finding is consistent with survival and hatching of embryos exposed to CPT at 48–72 hpf (Figure 1B and Figure 2).

**Figure 5:**
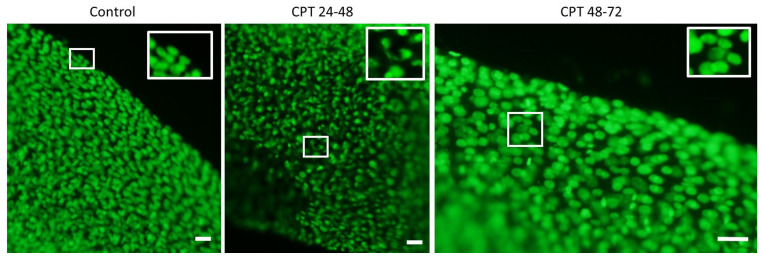
DNA staining in part of a sterlet fin at 7 dpf. The close-up images correspond to the squared area showing DNA aberrations. Images are representative of most embryos (*n* = 10). Scale bars correspond to 50 µm.

We stained total DNA in sterlet embryos fixed after control and CPT treatments (Figure 5). Images present part of the fin from hatchling larvae. Control embryos showed well-aligned nuclei of constant size and tightly packed nuclear DNA (Figure 5). DNA bridges and micronuclei were observed in embryos exposed to CPT at 24–48 hpf, in agreement with the results of the comet assay; embryos exposed to CPT at neuralization (48–72 hpf) were similar to controls.

### 2.3. DNA Damage Response in Sterlet Embryos

**Figure 6:**
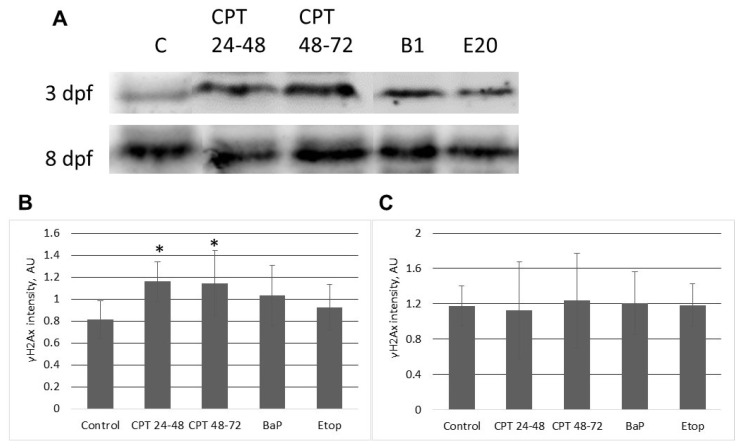
Western blotting analysis of phospho-H2AX (S139) (γ-H2AX) in proteins extracted from sterlet embryos at 3 and 8 dpf. (**A**) Representative images of western blots performed for six different cultures. “C”—control; “CPT 24–48”—10 nM CPT at 24–48 hpf; “CPT 48–72”—10 nM CPT at 48–72 hpf; “B1”—1 µM BaP; “E20”–20 µM etoposide. (**B**) Relative intensities of γ-H2AX bands at 3 dpf. (**C**) Relative intensities of γ-H2AX bands at 8 dpf. Values are expressed as means ± SEM (*n* = 6). Asterisks indicate significant differences compared to the control (Kruskal–Wallis test,* *p* < 0.1).

**Figure 7:**
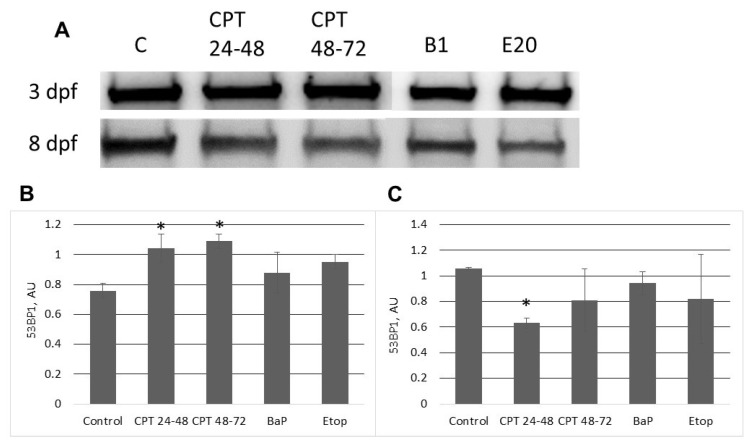
Western blotting analysis of 53BP1 protein level in sterlet embryos at 3 and 8 dpf. (**A**) Representative images of Western blots performed for three different cultures. “C”–control; “CPT 24–48”–10 nM CPT at 24–48 hpf; “CPT 48–72”—10 nM CPT at 48–72 hpf; “B1”—1 µM BaP; “E20”–20 µM etoposide. (**B**) Relative intensities of 53BP1 bands at 3 dpf. (**C**) Relative intensities of 53BP1 bands at 8 dpf. Values are expressed as means ± SEM (*n* = 3). Asterisks indicate significant differences compared to the control (Kruskal–Wallis test,* *p* < 0.1).

**Figure 8:**
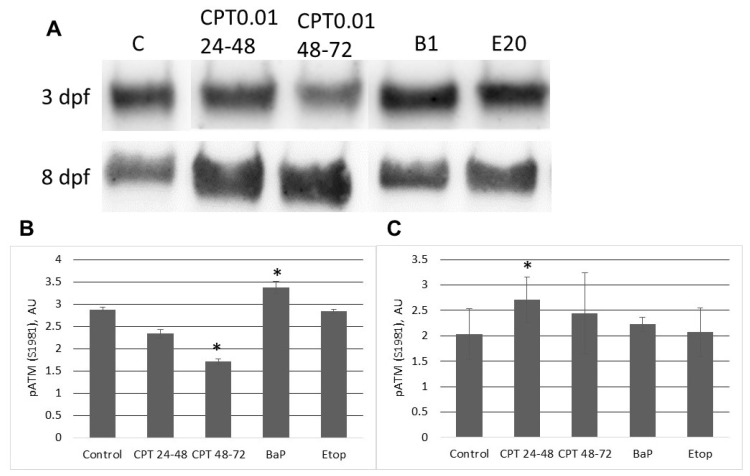
Western blotting analysis of phospho-ATM (S1981) in proteins extracted from sterlet embryos at 3 and 8 dpf. (**A**) Representative images of Western blots performed for 3 different cultures. “C”—control; “CPT 24–48”—10 nM CPT at 24–48 hpf; “CPT 48–72”—10 nM CPT at 48–72 hpf; “B1”—1 µM BaP; “E20”–20 µM etoposide. (**B**) Relative intensities of 53BP1 bands on 3 dpf. (**C**) Relative intensities of 53BP1 bands on 8 dpf. Values are expressed as means ± SEM (*n* = 3). Asterisks indicate significant differences compared to the control (Kruskal–Wallis test,* *p* < 0.1).

**Figure 9:**
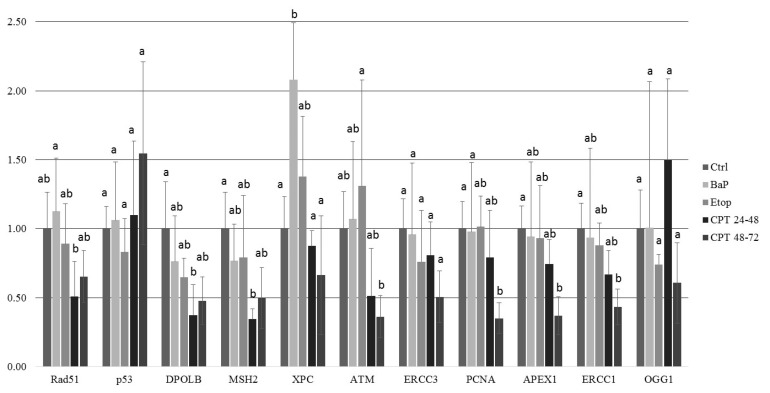
The mRNA expression DDR-related genes in 8 dpf sterlet embryos measured by qPCR. Embryos were exposed to BaP and etoposide continuously from fertilization to 8 dpf, and to CPT at 24–48 and 48–72 hpf. Results are represented as mean of at least four individual embryos. Error bars represent standard error of the mean. Letters indicate significant differences in gene expression between different treatments (Kruskal–Wallis test, *p* < 0.1).

Exposure to genotoxic compounds induced DNA damage in embryos with subsequent activation of DNA damage response pathways, as assessed with Western blotting and RT-qPCR (Figure 6, Figure 7, Figure 8 and Figure 9). Initially, we analyzed phosphorylation levels of H2AX (γ-H2AX) in 3- and 8-dpf embryos exposed to CPT, BaP, and etoposide (Figure 6A). The two time points were chosen since results of comet assays indicate that differences among treated and control embryos peak at these time points. Western blotting showed increased levels of γ-H2AX in 3-dpf embryos exposed to CPT at the gastrula stage. A significant increase of γ-H2AX was also observed in embryos exposed to CPT at neurulation (48–72 hpf). Further, continuous treatment with BaP or etoposide increased levels of γ-H2AX in 3-dpf embryos; however, these increases were not significant compared to control (*p* > 0.1, Figure 6B). Embryos collected at 8 dpf showed no differences in H2AX phosphorylation between control and treated embryos (Figure 6C).

We also analyzed another marker of DNA damage, 53BP1 (Figure 7A). Like γ-H2AX, 53BP1 expression was increased in 3-dpf embryos exposed to CPT compared to control (Figure 7B). Further, slightly elevated levels of the protein were observed in embryos exposed to BaP or etoposide (*p* > 0.1). 53BP1 decreased in CPT-treated embryos at 8 dpf compared to control (Figure 7C), in contrast to γ-H2AX results.

ATM kinase is a primary component of DNA damage response and checkpoint activation [5]. Level of autophosphorylation of ATM at S1981 significantly decreased in CPT-treated (48–72 hpf) embryos at 3 dpf (Figure 8A,B). However, embryos exposed to BaP showed a significant increase in phospho-ATM (S1981) (Figure 8B). After 8 days of development, embryos treated with CPT from 24–48 hpf displayed significantly increased ATM phosphorylation (Figure 8C). Levels of phospho-ATM (S1981) in embryos following CPT treatment from 48–72 hpf, and BaP, and etoposide treatments was like in control embryos.

Overall, the involvement of different DNA damage responses depends on the type of genotoxic insult. Interestingly, no DNA damage response was detected in embryos exposed to etoposide.

Further, we analyzed the expression of several genes involved in different pathways of DNA damage response and repair (Table 1; Figure 9). Gene expression was significantly reduced in embryos exposed to CPT which may indicate a delay in development. Expression of *rad51*, *dpolb*, and *msh2* was significantly downregulated in embryos treated with CPT at gastrulation (24–48 hpf). In contrast, levels of *p53*, *xpc*, *ercc3*, *ogg1* were like controls in all CPT-treated embryos. Exposure to CPT at neurulation led to significant downregulation of *atm*, *pcna*, *apex1*, *ercc1*. In BaP-treated embryos, the level of *xpc* mRNA was significantly increased (*p* < 0.1, Kruskal–Wallis test), whereas mRNA expression of other genes was similar to control. Etoposide did not induce changes in mRNA expression of selected genes.

## 3. Discussions

We present the first report on DNA damage and DNA repair induced in sterlet embryos by model genotoxicants. Genotoxicity in sterlet embryos was confirmed by the comet assay, DNA staining, and Western blotting with γ-H2AX and 53BP1 antibodies. DDR activity was evaluated by means of RT-qPCR and Western blotting with phospho-ATM antibody.

All tested compounds (BaP, etoposide, and CPT) induce DNA damage in sterlet embryos. However, the level of DNA damage and its effect on embryo survival and hatching rate varied. DNA damage induced by BaP and etoposide did not exceed 10% Tail DNA and was mostly repaired by 8 dpf. Conversely, exposure to 10 nM CPT before gastrulation (2–24 hpf) caused DNA fragmentation to reach 27% Tail DNA, which was lethal to the embryos. Lesser effects of BaP and etoposide could be attributed to differences in penetration through the embryo chorion. However, when etoposide was injected directly into the animal pole, it did not affect embryo survival. In contrast, we observed a significantly increased level of DNA fragmentation following embryo exposure to etoposide compared to the control. This fact indicates that etoposide can penetrate embryo chorion and induce DNA damage following simple exposure.

Interestingly, the control embryos also showed an increased level of DNA fragmentation at 2–3 dpf followed by gradual DNA repair at 5–8 dpf. A similar increase in DNA fragmentation in normally developing embryos was previously reported in zebrafish [16] and attributed to the activity of topoisomerase II (Top2) cleavage complexes. However, these authors did not evaluate levels of DNA damage at later stages of development and did not observe recovery of embryonic DNA. High levels of DNA fragmentation at early stages of embryo development could be related to rapid cell division [23]. After gastrulation, the cell cycle slows, cells acquire checkpoint activity and are effectively able to repair damaged DNA [26].

### 3.1. BaP Induces Alterations in DNA Structure and Activates the NER Pathway

This study aimed to assess the sensitivity of sterlet embryos toward different types of genotoxicity and to analyze DDR pathways activated in early stages of embryo development. Continuous exposure to BaP slightly increased DNA fragmentation and reduced survival and hatching rate of sterlet embryos. Further, BaP exposure was associated with increased ATM phosphorylation at 3 dpf and elevated *xpc* expression at 8 dpf. These results agree with previous studies that show teratogenic effects of BaP exposure on fish embryo development [12,13,27,28,29,30]. However, in contrast to model fish (zebrafish and medaka), sterlet embryos did not show a higher incidence of malformation following BaP exposure and effectively repaired DNA damage by 8 dpf. Prolongation of the cell cycle and increased checkpoint activity could assist with repair. Thus, sterlet embryos might repair altered DNA more effectively than fish species with more rapid embryo development. Further, reduced hatching rate could be due to the delay in development associated with checkpoint activation and DDR.

Overall, BaP does not appear to induce major DNA strand breaks in developing sterlet embryos, since no significant increase in % Tail DNA was recorded. However, it may form DNA adducts, in a manner similar to BaP-induced toxicity in human lung cancer [14]. A previous study on zebrafish embryos did not detect BaP-induced DNA damage with the comet assay; however, persistent DNA alterations were observed with qRAPD and AFLP techniques [27]. DNA adducts are further repaired by the NER pathway in human cell lines [14]. Similarly, we observed increased *xpc* gene expression and decreased DNA fragmentation at 8 dpf in sterlet embryos exposed to BaP. The protein encoded by *xpc* gene is a key component of the XPC complex that is vital in the early steps of global genome NER. Thus, in sterlet embryos, BaP induces the formation of DNA adducts, and increased ATM phosphorylation is followed by activation of the NER pathway.

### 3.2. The Effect of Etoposide on Sterlet Embryo

Exposure to etoposide (Top2 inhibitor) did not affect embryo survival and hatching rate, though DNA fragmentation was significantly increased at 2–3 dpf. This result is consistent with previous studies on fish embryos [16,31,32]. Top2 inhibitors can induce DNA damage without affecting embryo survival in zebrafish [16]. Interestingly, we did not observe significant changes in H2AX, ATM phosphorylation, or mRNA expression levels. However, the level of 53BP1 was slightly increased compared to control at 3 dpf (*p* > 0.1, Kruskal–Wallis test). Similarly, Muslimović et al. [33] showed that only a small fraction of all etoposide-induced DSBs activate H2AX phosphorylation and induce toxicity in human cell lines. Ninety percent of DSBs produced by etoposide are held in a Top2-linked complex that is not recognized by ATM or other DNA damage recognition systems. This feature of etoposide action explains the increased level of DNA fragmentation without an increase of γ-H2AX and ATM phosphorylation. However, the effects of formation of DSB-Top2 complexes on embryo development are not clear. Further studies are needed to elucidate the consequences of DNA damage induced by etoposide. Our results indicate that at later stages of development etoposide exposure does not induce DNA fragmentation. This finding might be attributed to changing expression patterns of Top2 at different stages of development [34].

### 3.3. The Stage-Dependent Effect of CPT on Sterlet Embryo Development

Inhibition of DNA topoisomerase I (Top1) with CPT was lethal for sterlet embryos in early stages of development (before gastrulation). Similarly, previous studies demonstrate that Top1 inhibition is detrimental to post-MBT zebrafish embryos at the same developmental stage [16,26]. In fish embryos, gastrulation is a critical phase, when cells acquire the ability to enter apoptosis [26]. Treatment with CPT before gastrulation causes zebrafish embryo death at the end of gastrulation (10 hpf) [26]. During the gastrula stage, authors observed that CPT treatment caused arrest of proliferation, followed by apoptosis in some fraction of cells. If a sufficiently high fraction of embryonic cells were postmitotic at the time of treatment, embryos survived for hours. In our study, embryos that have been exposed to CPT during gastrulation and neurulation showed high survival and hatching rate. Thus, both Top1 and Top2 inhibitors showed time-dependent effects in fish embryos. However, in contrast with etoposide, the stage-dependent effect of CPT is attributed to differences in cell cycle and cell proliferation at different stages, rather than changes in the pattern of topoisomerase expression [34].

In contrast to zebrafish embryos, which survive only several hours following exposure to CPT, sterlet embryos, exposed to CPT at gastrulation or neurulation, survived up to several days and showed signs of recovery. Activation of DDR pathways was indicated by increased levels of γ-H2AX and 53BP1 at 3 dpf. Interestingly, levels of phospho-ATM (S1981) decreased at the same time point. H2AX can also be phosphorylated by other phosphoinositide 3-kinase related protein kinases (PIKKs) such as ATM- and Rad3-related (ATR), and DNA-dependent protein kinase [35]. Therefore, in sterlet embryos treated with CPT at gastrulation and neurulation, H2AX phosphorylation was mediated by kinases other than ATM [36]. However, further studies are needed to confirm the mechanism of H2AX phosphorylation in response to CPT exposure.

At 8 dpf, the level of γ-H2AX was the same as in control, but phospho-ATM (S1981) increased, and 53BP1 decreased in embryos exposed to CPT at gastrulation (24–48 hpf). Exposure to CPT at neurulation (48–72 hpt) did not alter levels of γ-H2AX, phospho-ATM (S1981), and 53BP1 compared to control. This observation correlates with DNA integrity, viability, and hatching rate. However, we observed decreased mRNA expression of several genes related to DNA repair. The amount of mRNA extracted from extracted from CPT 24–48 embryos was lower than in controls. Therefore, it can be suggested that embryos were delayed and, hence, the level of transcription and protein expression (53BP1) was also lower. Previous results that showed checkpoint activation in fish embryos treated with CPT are consistent with our finding [26]. Furthermore, checkpoint activation is typically associated with increased ATM activity [5]. Nevertheless, further studies are needed to confirm that checkpoint activity in sterlet embryos is involved in CPT-induced responses.

## 4. Conclusions

This study presents the first screening of DNA damage response pathways utilized by sterlet embryos. These embryos are generally more resistant to genotoxicants than embryos of zebrafish. Further, early stages of development (before neurulation) are more susceptible to genotoxic stress than later stages. It could be due to prolonged cell cycle at later stages, as well as reduced cell proliferation and DNA replication. The presented pieces of evidence suggest that sterlet embryos could be used as an alternative or complementary model for studies on DNA damage responses in fish.

The current study confirms our hypothesis that mechanisms of DDR are highly effective in sterlet embryos. They can recover from BaP-induced toxicity, possibly through the NER pathway. Embryo viability and hatching seem to be unaffected by etoposide exposure; however, it is possible that etoposide induces the formation of DSB-Top2 complexes, which are not recognized by DDR pathways. Besides, sterlet embryos can recover from as much as 15% DNA fragmentation following CPT exposure. Nevertheless, further studies are needed to elucidate the spatial-temporal distribution of DDR proteins in developing sterlet embryos and their deployment during normal embryonic development.

Notably, only a few DDR-related antibodies have been tested in fish [4]; and the current study is the first report on phospho-ATM (S1981) in sterlet. We observed some differences between phospho-ATM (S1981) and γ-H2AX levels in response to genotoxicants. Thus, other PIKKs, such as ATR, might be involved in DNA damage response in sterlet embryos.

## 5. Materials and Methods

### 5.1. Ethics

Gametes were collected from sterlet during the natural spawning period. During broodstock handling, fish were anesthetized using 0.07 mL^−1^ of clove oil. All experiments were performed on fish embryos before reaching an independently feeding larval form, no special approval of the local ethical committee was needed according to the law to protect animals against maltreatment (no. 246/1992 Collection of Laws).

### 5.2. Animals

The sterlet (BW, 0.5–2 kg; 12 females, 9 males) were reared in the aquaculture facility of the Research Institute of Fish Culture and Hydrobiology at the University of South Bohemia, Vodňany, Czech Republic. In order to induce ovulation, female fish were injected with carp pituitary extract powder dissolved in 0.9% (*w/v*) NaCl solution at an initial dose of 0.5 mg/kg of body weight. The second injection with carp pituitary powder (4.5 mg/kg of body weight) was performed 12 h after the first injection. Oocytes were collected 18–20 h after the second injection through a minimally invasive incision of the oviduct. Sperm production in males was induced with an intramuscular injection of carp pituitary extract powder (4.0 mg/kg of body weight) dissolved in 0.9% (*w/v*) NaCl solution. After 48 h post-injection, sperm was collected from the urogenital papilla with a catheter. Sperm was stored at 4 °C in separate cell culture containers (250 mL) until use. Eggs were fertilized with sperm activated in dechlorinated tap water at 15 °C for 2 min, and the stickiness of fertilized eggs was removed by 3× washings with 0.1% tannic acid solution for 1 min. Identification of early developmental stages was based on studies of *Acipenser baerii* and *Acipenser güldenstädti* [24,37]. Embryos were incubated in Petri dishes with dechlorinated tap water at 16 °C, and each Petri dish contained approximately 50 embryos.

### 5.3. Reagents

Etoposide (inhibitor of topoisomerase II) (CAS Number 33419-42-0), benzo[a]pyrene (CAS Number: 50-32-8), and camptothecin (inhibitor of topoisomerase I) (CAS Number: 7689-03-4) were purchased from Sigma-Aldrich Co. (St Louis, MO, USA). Mouse monoclonal antibodies [9F3] to gamma H2A.X (phospho S139), mouse monoclonal antibodies to phospho-ATM (S1981), and polyclonal 53BP1antibodies produced in rabbits were purchased from Abcam (Cambridge, UK).

### 5.4. Experimental Design

Concentrations of compounds were selected based on available literature data and preliminary experiments [13,16]. We analyzed the effects of BaP on sterlet embryo development over a concentration range of 0.5–5 µM (Table S1). One micromolar was chosen as the highest tested concentration soluble in water. CPT showed high toxicity to sterlet embryos in preliminary experiments (5–100 nM: Table S1). Ten nanomolars CPT was selected as the lowest dose that showed an effect on embryo development.

All compounds were dissolved in DMSO as stock solutions. Immediately before the treatment, stock solutions were diluted 100x as working solutions. Working solutions were added to the embryo culture medium (dechlorinated tap water) at a 1:1000 ratio to produce required concentrations in medium and 0.1% DMSO (*v/v*). Working solutions were prepared daily. The same amount of DMSO (0.1% in embryo culture medium) was used as solvent control.

For each treatment, a group of 50 fertilized embryos at the 2-cell stage was transferred to a Petri dish. Each dish contained either vehicle control or a tested compound in a total of 30 mL of dechlorinated tap water. Embryos were exposed to 1 µM BaP or 20 µM etoposide continuously from 2-cell stage until hatching, or to 10 nM CPT for 24 h during one of three stages: (1) from 2 to 24 hpf (2–24 hpf; blastula); (2) from 24 to 48 hpf (gastrulation); (3) from 48 to 72 hpf (neurulation). CPT was replaced with fresh water after 24 h. Embryos were held in separate Petri dishes in dechlorinated tap water with 0.1% DMSO before exposure. Water was changed daily, and dead embryos were continuously removed. Each experiment was repeated six times.

Embryos were examined daily under a stereomicroscope (NSZ-608T, Nanjing Jiangnan Novel Optics Co., Ltd., Nanjing, China) and counted until hatching (8 dpf). Throughout embryogenic development, the following parameters were analyzed:

1. embryonic viability, % [number of dead embryos/number of fertilized eggs × 100];
2. hatching rate, % [number of hatched larvae/number of fertilized eggs × 100];
3. occurrence of malformations.

The embryo phenotypes were observed under a microscope (NSZ-608T, Nanjing Jiangnan Novel Optics Co., Ltd., Nanjing, China) and imaged at ×2.5 magnification. A minimum of 150 embryos were analyzed per treatment.

### 5.5. Microinjection

Glass microcapillaries were drawn on a needle puller (PC-10; Narishige, Tokyo, Japan), and solution of 20 µM etoposide, 0.2 M KCl, and 1% Rhodamine dextran (MW 10,000) was Microinjected into embryos. Rhodamine was used for visual control of the delivery of solution during injection via the appearance of red color under the stereomicroscope and again during sorting under a fluorescence stereomicroscope. Microcapillaries loaded with solution were mounted on a micromanipulator (MN-153, Narishige, Japan) attached to a pneumatic injector (FemtoJet^®^ 4×, Eppendorf, Hamburg, Germany). Non-dechorionated embryos were placed in a Petri dish coated with 1% agar. Tips of the microcapillaries were carefully broken with forceps. The duration and pressure of injection pulses were determined using the oil droplet method and kept constant over the experiment while ~10 nL was injected into the animal pole of each embryo.

### 5.6. Comet Assay

DNA integrity was assessed using alkaline comet assays, or single-cell gel electrophoresis assays, following previously described methods [38] with minor modifications. Briefly, embryos at different stages were transferred into 1.5 mL tubes (3–4 embryos in each) with 1× PBS on ice. Embryos were mechanically disintegrated and centrifuged at 1000× *g* for 3 min. The pellets containing embryonic cells were washed with 1 mL of PBS, centrifuged at 1000× *g* for 3 min, and resuspended in 1 mL of PBS.

**Figure 10:**
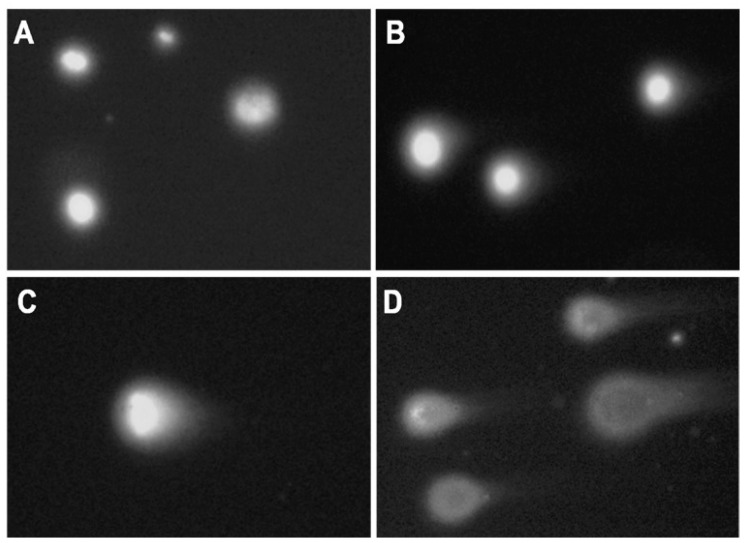
DNA fragmentation in sterlet embryos analyzed by comet assay. Examples from the comet assay of *Acipenser ruthenus* embryos exposed to different treatments (**A**) control, (**B**) etoposide 20 µM, (**C**) CPT 10 nM, (**D**) CPT 50 nM. Relative changes in DNA fragmentation are represented by an increasing amount of DNA in the comet tail.

Microscope slides (OxiSelectST; Cell Biloabs, INC., San Diego, CA, USA) used for the assay were prepared as follows: resuspended samples (200 µL) were mixed with 700 µL 0.7% NuSieve GTG low melting point agarose (OxiSelectST; Cell Biloabs, INC., San Diego, CA, USA). Forty microliters of this mixture was placed on the slide, and the agarose was allowed to solidify at 4 °C. Further, slides were immersed in lysis buffer (2.5 M NaCl, 100 mM EDTA, 10 mM Tris, 1% Triton X-100, 10% DMSO, pH 10) and incubated overnight at 4 °C. After cells lysis, the buffer was removed, and slides were placed in a horizontal gel tank filled with freshly prepared electrophoresis buffer (90 mM Tris Base, 90 mM Boric acid, 2.5 mM EDTA). Electrophoresis was performed for 20 min at 35 V and 170 mA. Following electrophoresis, slides were washed three times with pre-chilled DIH2O for 2 min. Washed slides were dehydrated with 70% ethanol, and air-dried for storage at 4 °C. Before the analysis, 50 µL of PBS containing DAPI/Antifade Staining Solution (Sigma-Aldrich Co., St Louis, MO, USA) was added to the slides for agarose rehydration and DNA staining. Slides were analyzed using an Olympus BX50 fluorescence microscope at 20× magnification (Figure 10). At least 30 cells were scored for each sample. The images were analyzed with CometScore image analysis software (TriTek Corporation, Sumerduck, VA, USA). Tail length (measured from the middle of the head to the end of the comet tail) and % Tail DNA (the content of DNA in comet tails) were measured. The per cent DNA in the tail was calculated using the following formula:

%Tail DNA = 100 × Tail DNA intensity/Cell DNA intensity

### 5.7. Western Blotting Analysis

Embryos at different stages of development were dechorionated with forceps and mechanically dissociated in protein extraction buffer (8 M Urea, 2 M Thiourea, 4% CHAPS, 0.1% *w/v* Triton X-100, 100 mM dithiothreitol) containing phosphatase inhibitors (1 mM sodium orthovanadate, 50 mM EDTA, 1 mM okadaic acid) and protease inhibitors (100 mM PMSF, 1 mg/mL pepstatin A, 5 mg/mL leupeptin). Proteins were extracted from at least four embryos per condition. The analysis was repeated three times for each experimental condition. Bradford assay was used to determine protein concentrations in each sample. The absorbance at 595 nm was measured with Biobase-EL10A microplate reader (Biobase, Shandong, China).

For SDS-PAGE, proteins were mixed with sample buffer containing 65 mM Tris, 10% (*v/v*) glycerol, 2% (*w/v*) SDS, 5% (*v/v*) beta-mercaptoethanol, and 1% bromophenol blue. Those samples were then heated for 3 min at 95 °C. Proteins were separated on a 10% acrylamide gel using a Bio-Rad Mini-PROTEAN vertical electrophoresis system; 25 µg of protein was loaded into each lane.

Following SDS-PAGE, proteins were electrically transferred from gels to polyvinylidene difluoride (PVDF) membranes. Total protein content (loaded and transferred) was controlled before immunodetection by staining the membrane with 0.1% (*w/v*) Coomassie Brilliant Blue R-250 in isopropanol. After staining, membranes were scanned with imaging system Fusion Solo 7S Edge (Vilber Lourmat, Collégien, France). After imaging, membranes were destained in acetic acid/ethanol/water (1:7:2). Destained membranes were washed with PBST (0.1% *v/v* Tween-20, PBS) three times at 20 °C. Membranes were blocked by incubation with 5% (*w/v*) bovine serum albumin (BSA) in PBST for 1 h at 20 °C, then incubated for 16 h at 4 °C with antibodies to either gamma H2A.X (phospho S139), phospho-ATM (S1981) or 53BP1 diluted 1:3000 in 5% *w/v* BSA in PBST. Membranes were then washed and incubated with horseradish peroxidase-conjugated goat anti-rabbit or goat anti-mouse IgG (1:10,000 in 3% *w/v* BSA-PBST) for 1 h at 20 °C. Reacted proteins were detected with ECL-plus (GE Healthcare, Danderyd, Sweden) using Fusion Solo 7S Edge (Vilber Lourmat, France). The intensity of each band was measured using ImageJ (NIH, Bethesda, MD, USA) and normalized against total protein content in each sample. Total protein staining was used as a loading control (Figure S1).

### 5.8. RT-qPCR

The genes studied were previously reported to be expressed in developing fish embryos [4,39], and Table 1 shows primers used for RT-qPCR. Primers were designed with Primer3 software (https://primer3.org/) based on nucleotide sequences from sterlet transcriptomes (NCBI, Table 1).

Embryos for RT-qPCR were collected and immediately transferred to a −80 °C freezer. Total RNA extraction used 1 mL of Trizol (Sigma-Aldrich) following the manufacturer’s protocol, then precipitated with LiCl. Total RNA concentration was determined with a NanoDrop 2000 (ThermoScientific, Wilmington, DE, USA), and the quality of RNA was assessed using a fragment analyzer (Advanced Analytical).

After extraction, 1 µg of isolated total RNA was reverse transcribed into cDNA with SuperScript III Reverse transcriptase kit (Invitrogen). We prepared a mixture of RNA with 0.5 µL of oligo-dT and random hexamers (mixture 1:1, 50 µM each), 0.5 µL of dNTPs (10 mM each), 0.5 µL of the spike (TATAA Universal RNA Spike, TATAA Biocenter, Göteborg, Sweden), and water to a total volume of 6.5 µL. This mixture was incubated for 5 min at 75 °C, 20 s at 25 °C, and 4 °C for 1 min. After incubation, we added 100 U of SuperScript III enzyme, 20 U of RNaseOUT (Invitrogen, Life Technologies, Carlsbad, CA, USA), 0.5 µL of DTT, and 2 µL of 5× first-strand synthesis buffer to a final volume of 10 µL. The mixture was then incubated at 25 °C for 5 min, 50 °C for 60 min, 55 °C for 15 min, and 75 °C for 15 min. After incubation, 90 µL of distilled H_2_O were added, and cDNA was stored at −20 °C. qPCR mix was composed of 5 µL of TATAA SYBR^®^ GrandMaster Mix (TATAA Biocenter), 0.5 µL of forward and reverse primers mix (mixture 1:1, 10 µM each), 2 µL of cDNA and distilled H_2_O. The final volume of qPCR mix was 10 µL. qPCR was performed on a CFX384 cycler system (Bio-Rad Laboratories, Hercules, CA, USA). The following PCR conditions were used: initial denaturation at 95 °C for 2 min, 40 repeats of denaturation at 95 °C for 15 s, annealing at 60 °C for 20 s, and elongation at 72 °C for 20 s. Melting curve analysis was performed to test the specificity of amplification. Total RNA concentration was used for normalization in RT-qPCR. Data were analyzed using Excel and delta Cq approach.

### 5.9. Embryo DNA Staining

Embryos at hatching (7 dpf) were fixed with ice-cold 100% ethanol and stored at −20 °C. Ethanol was removed for DNA staining, and embryos were rehydrated with successive washing in 75, 50, 25, and 0% ethanol in PBS. Subsequently, samples were incubated in PBST containing 3% BSA for 1 h at 20 °C on a shaker. Embryos were stained with 1 µg/mL SYBR Green in PBST for 1–2 h at 20 °C on a shaker. Finally, embryos were washed twice with PBS, mixed with 1% melted agarose dissolved in PBS, and transferred to a glass slide. The DNA aberrations were imaged using a fluorescent microscope, Olympus BX50, at 10× magnification.

### 5.10. Statistical Analysis

Normality and homogeneity of variance of all data were first assessed with Kolmogorov and Bartlett tests, respectively. Values for comet assay are expressed as means ± SEM. Statistical comparison was made by one-way ANOVA followed by Tukey’s HSD test (significance level of 0.05). Values for viability, hatching rate, Western blots, and RT-qPCR are expressed as means ± SEM and analyzed using the Kruskal–Wallis test. Analyses of viability, hatching rate, and the comet assay were performed using a significance threshold of 0.05. In contrast, analysis of RT-qPCR and Western blotting was performed using a significance threshold of 0.1 with STATISTICA 13.0 software for Windows.

## Figures and Tables

**Table 1:** List of primers used in RT-qPCR.

Gene	NCBI Reference	Sequence 5′–3′	Encoded Protein Function	DDR Pathway
*rad51*	XM_034041201	TTGCTGAAAGGTACGGGCTA TCGTAGAAACCTGGCCAAGT	Binds to single and double-stranded DNA and catalyzes the recognition of homology and strand exchange between homologous DNA partners to form a joint molecule between a processed DNA break and the repair template.	HR
*tp53*	XM_034915522	CGGGCCTCAATAAGCTGTTC ACAGGGCCTTCGTTGTTTTC	Induces growth arrest or apoptosis, involved in cell cycle regulation.	Checkpoint
*dpolb*	XM_033996470	GGAGAGGTGCAGAGTCAAGT CCGAGCCCTCATCATCATCT	Repair polymerase that plays a key role in base-excision repair.	BER
*msh2*	XM_034003209	ATCCCCAATGACGTGACCTT CAATGCTGATCTCCGCTGAC	Forms two different heterodimers, which binds to DNA mismatches thereby initiating DNA repair.	DNA mismatch repair (MMR)
*xpc*	XM_034906644	TTGGCTGTGTTCGAATGCAA TCTGCTTGCTCATTCTCCCA	Involved in global genome nucleotide excision repair (GG-NER) by acting as damage sensing and DNA-binding factor component of the XPC complex.	NER
*atm*	XM_034011595	CAAGTGTCACCGTCAAGCAA CAGCATGGACATAACACCCG	Serine/threonine protein kinase which activates checkpoint signaling upon double strand breaks, apoptosis and genotoxic stresses, thereby acting as a DNA damage sensor.	Sensing/Checkpoint
*ercc3*	XM_034001899	GCGACACGTCCTTTGATCTC CCATGCGAGTGATCACCTTG	ATP-dependent 3′–5′ DNA helicase, component of the general transcription and DNA repair factor IIH (TFIIH) core complex.	NER
*pcna*	XM_033996605	ACTGATGGACCTGGATGTGG ACAGCCTCCTCCTCTTTGTC	Plays a key role in DDR by being positioned at the replication fork to coordinate DNA replication with DNA repair. Acts as a loading platform to recruit DDR proteins that allow completion of DNA replication after DNA damage.	BER
*apex1*	XM_034916619	ATTGTGCGCTGTGTGGATTT TCCACTCTCGCTTCGTTCTT	Functions as an apurinic/apyrimidinic (AP) endodeoxyribonuclease in the BER pathway of DNA lesions induced by oxidative and alkylating agents.	BER
*xrcc1*	XM_034917452	TCAACGGACGAGAACACAGA AATCCTGGGCTGTGATGACA	Involved in mediating the assembly of DNA break repair protein complexes	BER
*ogg1*	XM_034906453	GCCCAACGGAATTCTGACTC CACTGTAACGCTGGGGTCTA	DNA repair enzyme that incises DNA at 8-oxoG residues.	BER

HR—homologous recombination; BER—base excision repair; NER—nucleotide excision repair.

## Supplementary Materials

The following are available online at https://www.mdpi.com/1422-0067/22/1/6/s1.

## Abbreviations

DDR	DNA damage repair
SSB	Single-strand break
DSB	Double-strand break
BER	Base excision repair
NER	Nucleotide excision repair
HR	Homologous recombination
NHEJ	Non-homologous end-joining
ATM	Ataxia telangiectasia mutated kinase
BaP	Benzo[a]pyrene
CPT	Camptothecin
hpf	Hours post-fertilization
dpf	Days post-fertilization
