# Supplementary material for: Ancient Sturgeons Possess Effective DNA Repair Mechanisms: Influence of Model Genotoxicants on Embryo Development of Sterlet, Acipenser ruthenus
Source: Int J Mol Sci. 2020 Dec 22;22(1):6. doi: 10.3390/ijms22010006 (PMC7792610; doi:10.3390/ijms22010006)
Supplement: Supplementary file 1 [file ijms-22-00006-s001.pdf]

International Journal of *Molecular Sciences*

MDPI

Supplementary

Ancient Sturgeons Possess Effective DNA Repair Mechanisms: Influence of Model Genotoxicants on Embryo Development of Sterlet, *Acipenser Ruthenus*

Ievgeniia Gazo [1,*], Roman Franěk [1], Radek Šindelka [2], Ievgen Lebeda [1], Sahana Shivaramu [1], Martin Pšenička [1] and Christoph Steinbach [1]

[1] South Bohemian Research Center of Aquaculture and Biodiversity of Hydrocenoses, Faculty of Fisheries and Protection of Waters, University of South Bohemia in Ceske Budejovice, Zátiší 728/II, 389 25 Vodňany, Czech Republic; franek@frov.jcu.cz (R.F.); ilebeda@frov.jcu.cz (I.L.); sahana.s92@gmail.com (S.S.); psenicka@frov.jcu.cz (M.P.); steinbach@frov.jcu.cz (C.S.)

[2] Laboratory of Gene Expression, Institute of Biotechnology—Biocev, Academy of Science of the Czech Re public, 252 50 Vestec, Czech Republic; radek.sindelka@ibt.cas.cz

* Correspondence: gazo@frov.jcu.cz; Tel.: +420 38777 4607

Citation: Gazo, I.; Franěk, R.; Šindelka R..; Lebeda, I.; Shivaramu, S.; Pšenička M.; Steinbach, C. Ancient Sturgeons Possess Effective DNA Repair Mechanisms: Influence of Model Genotoxicants on Embryo Development of Sterlet, *Acipenser Ruthenus*. *Int. J. Mol. Sci.* **2021**, *22*, 6. https://doi.org/10.3390/ijms22010006

Received: 1 December 2020
Accepted: 19 December 2020
Published: 22 December 2020

Publisher's Note: MDPI stays neutral with regard to jurisdictional claims in published maps and institutional affiliations.

Copyright: © 2020 by the authors. Submitted for possible open access publication under the terms and conditions of the Creative Commons Attribution (CC BY) license (http://creativecommons.org/licenses/by/4.0/).

Table S1. Sterlet embryo viability and hatching rate following exposure to different concentrations of BaP (0.5, 1, 5 μM), etoposide (1, 5, 10, 20 μM), and CPT (5, 10, 50, 100 nM).

Treatment	Live,%	Hatched,%
Control	91	76
BaP 0.5μM	94	73
BaP 1μM	84	59
BaP 5μM	68	58
Etop 1μM	84	77
Etop 5μM	89	79
Etop 10μM	82	71
Etop 20μM	88	75
CPT 5nM	88	71
CPT 10nM	75	21
CPT 50nM	0	0
CPT 100nM	0	0

Embryos were exposed to BaP and etoposide from 2 hpf till 8 dpf; and to CPT from 24 till 48 hpf. All results are presented at 8 dpf. Results represent mean of two independent experiments, number of embryos N = 50.

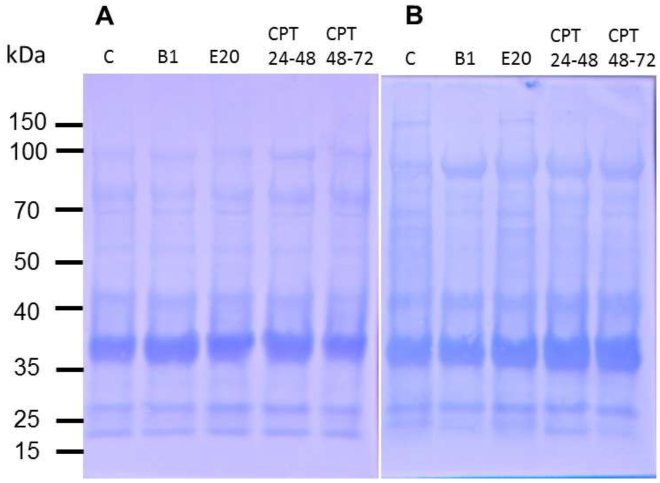

Figure S1. Total protein profile of sterlet embryos on (A) 3 dpf; (B) 8 dpf. Proteins were stained with 0.1% (w/v) Coomassie Brilliant Blue R-250 in isopropanol. "C"—control; "CPT 24–48"—10 nM CPT at 24–48 hpf; "CPT 48–72"—10 nM CPT at 48–72 hpf; "B1"—1 μM BaP; "E20"–20 μM etopo-side. Molecular weight marker (kDa) is on the left.
